# Precision cell-free DNA extraction for liquid biopsy by integrated microfluidics

**DOI:** 10.1038/s41698-019-0107-0

**Published:** 2020-02-24

**Authors:** Hoyoon Lee, Chanhee Park, Wonhwi Na, Kyong Hwa Park, Sehyun Shin

**Affiliations:** 1 School of Mechanical Engineering, Korea University, Seoul, 02841 Republic of Korea; 2 Nano-Biofluigonstic Research Center, Korea University, Seoul, 02841 Republic of Korea; 3 Division of Oncology/Hematology, Department of Internal Medicine, Korea University College of Medicine, Seoul, 02841 Republic of Korea

**Keywords:** Molecular medicine, Breast cancer, Nanobiotechnology, Prognostic markers

## Abstract

Cell-free DNA (cfDNA) has been implicated as an important biomarker in cancer management. Thus, efficient techniques for cfDNA extraction are necessary for precision medicine. We developed a centrifugation-free cfDNA extraction microfluidic chip capable of extracting cfDNA from plasma samples through microfluidic circuits within 15 min under vacuum pressure using an immiscible solvent. The microfluidic chip had excellent performance that was comparable to the most widely used commercial product (QIAamp kit) in terms of extraction efficiency, purity, and quality of DNA samples. The microfluidic chip was validated for the continuous monitoring of *HER-2* type breast cancer and was able to successfully detect a point mutation in phosphatidylinositol-4,5-bisphosphate 3-kinase (*PIK3CA*) during severe liver metastasis. The chip effectively eliminates the repetitive centrifugation processes and dramatically shortened the sample preparation time. The proposed platform could facilitate the development of a sample-to-answer system for use in liquid biopsy of cancers.

## Introduction

Technical advances in molecular diagnostics based on nucleic acids have advanced the use precision medicine in clinical applications.^1–3^ Among nucleic acids, DNA that freely circulates in the bloodstream (cell-free DNA, cfDNA) is a non-invasive and real-time biomarker of cancer that is useful for diagnosis, prognosis, treatment selection, and monitoring of tumor burden.^3–6^ Liquid biopsy, which detects circulating tumor DNA (ctDNA) in blood, has been investigated as an alternative to tissue biopsy that can overcome limitations such as sampling bias, intratumoral heterogeneity, and difficulty in repetitive sample extraction.^7–9^

The natural characteristics of cfDNA make it a challenging analyte to extract. One of the main hindrances in extracting cfDNA is its considerably low concentration in the plasma (average 30 ng/mL, range 1.8–44 ng/mL).^10,11^ The average concentration of cfDNA in cancer patients tends to increase to 180 ng/mL,^12–15^ but this still remains difficult to detect. While ctDNA can provide tumor genetic information, but it constitutes only 0.01% of total cfDNA (0.01%).^16–19^ Furthermore, large amounts of interfering cfDNA can make it difficult to accurately detect rare mutant targets. Furthermore, cfDNA should be purified as soon as possible, since it is short (~180 bp) and fragmented, with a short half-life (16–150 min).^4,6,20,21^ As any loss of target analytes can result in the misdiagnosis of cancer, an accurate, easy, and rapid method of sample preparation in liquid biopsy is required to reduce sample preparation error. Since the extraction process precedes the polymerase chain reaction (PCR) process, the recovery rate of cfDNA in the extraction process will affect the precision of the subsequent process. Thus, precision liquid biopsy requires precision cfDNA extraction.

Currently, cfDNA extraction is done using columns or magnetic beads, a phenol-chloroform-based method, and a filtration-based method.^6^ Among these methods, commercial spin column kits containing silica membranes have been widely used in clinical applications.^22–24^ In the silica-based solid phase extraction methods, nucleic acids can bind to the silica surface under high chaotropic salt conditions and can be detached at low salt concentration.^25,26^ Although the spin column method provides high yield and high purity of DNA, it requires a benchtop centrifuge operating at an extremely high centrifugal force and has an inconsistent workflow, with the use of various solvents or drying of the washing reagent remaining inside the silica membrane being required. Furthermore, this method involves manual handling, like frequent sample transfer to other tubes and removing and replacing spin-columns for centrifugation. These steps increase both the complexity of the procedure and the risk of cross contamination, require a skilled operator, are expensive, and prolong to time of the procedure.^27^

Microfluidic applications have advanced nucleic acid extraction. Various solid phase microfluidic extractions have been proposed using either an extended surface area in microchannels,^28–30^ miniaturized fluidic chips including silica membranes,^31,32^ or silica beads.^33–35^ We previously introduced a pressure and immiscibility-based extraction (PIBEX) method for centrifugation-free extraction of cfDNA with a silica membrane under vacuum pressure using an immiscible liquid, such as mineral oil. However, microfluidic integration and automatic fluid control were not realized.^36^ Recently, a lab-on-a-disc system containing silica-coated beads^37^ and surface modification using a non-chaotropic agent, dimethyl dithiobispropionimidate (DTBP),^38^ were successfully used for cfDNA extraction and a cancer monitoring test. However, several aspects of the system, such as full automation, large work capacity for simultaneous multiple extraction in a single operation, complete incorporation of separate experimental steps, and ease of use with low total cost, needed to be further developed for potential clinical use.

Here, we developed the integrated microfluidic PIBEX chip, which permits easy, rapid, and centrifugation-free cfDNA extraction from blood plasma. In this study, we compared the performance of the PIBEX chip to the conventional gold standard QIAGEN spin column kit in terms of cfDNA extraction yields, purity, and quality tests. Feasibility tests to identify the presence of ctDNAs was confirmed by digital droplet PCR (ddPCR) using a cfDNA reference standard set. As a proof-of-concept study, we utilized the PIBEX chip for a case study of liquid biopsy in *HER-2* metastatic breast cancer. In a serial monitoring study, we observed a significant fraction change of the H1047R mutation in phosphatidylinositol-4,5-bisphosphate 3-kinase (*PIK3CA*) during metastasis of the breast cancer to the liver using the ddPCR assay.

## Results

### Integration of cfDNA extraction process into a microfluidic chip

**Fig. 1:**
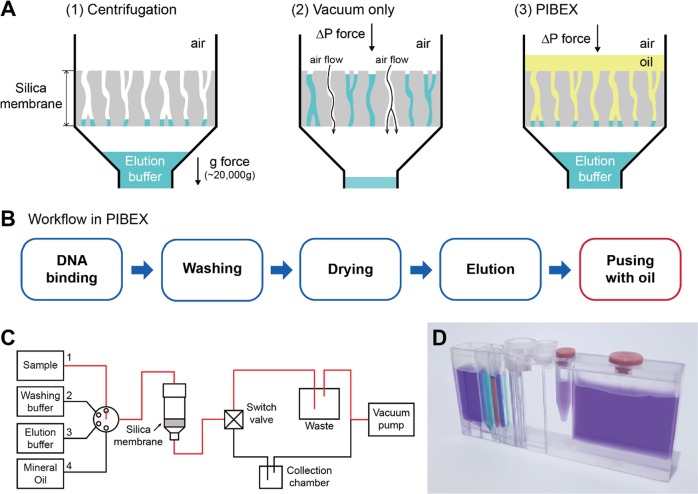
Integrated Microfluidics (PIBEX) for cfDNA extraction. **a** Comparison of cfDNA extraction methods. The schematic illustrates mechanisms of extracting residual eluents in silica membrane using centrifugation, vacuum, and immiscible fluid and vacuum (PIBEX), respectively. This work is reproduced under CC BY 4.0/partial use and word modifications from the original.^36^ **b** Workflow of the proposed microfluidic process of cfDNA extraction. The microfluidic PIBEX method is identical to the conventional spin column method, except for the last additional phase of “pushing with oil,” which collects eluents that have remained inside the silica membrane using an immiscible solvent. **c** Schematic of the flow channel at the DNA binding step. The flow pathway is selectively connected from sample chamber to collection chamber, as depicted by a red line. **d** Photograph of an integrated microfluidics for cfDNA extraction into PIBEX chip.

In silica membrane-based DNA extraction, a force that is sufficient to permit flow through membranes and a high recovery rate of the elution buffer is crucial for the efficient extraction of DNA. Silica membrane-based centrifugal force extraction is widely used for cfDNA extraction.^10,11^ However, due to the surface tension in the microscale environment, most of the residual liquid can be collected even after centrifugation at an extremely high g-force of approximately 12,000–20,000 × g (Fig. 1a). Compressed air or a vacuum can be used as the driving force for flow through a silica membrane. Even then, a large amount of liquid inside the membrane cannot be pushed out.

In our previous study,^36^ we proposed the PIBEX concept, in which an immiscible liquid is flowed into the silica membrane to alter the dominant surface tension in the microscale environment of the membrane. Using vacuum pressure and the immiscible fluid, all buffer liquid captured in a silica membrane was easily pushed out and successfully extracted. The PIBEX method can replace the use of centrifugal force. Mineral oil was chosen as a proper immiscible solvent, since it does not mix with the elution buffer and does not interact with DNA amplification during PCR. Mineral oil is commonly used in PCR experiments to minimize evaporation and to prevent change in buffer composition, which can affect enzyme activity.^39–41^

The cfDNA extraction using the PIBEX chip is illustrated in Fig. 1. The workflow proceeds with DNA binding, washing, drying, eluting, and pushing with oil (Fig. 1b). A plasma (0.5 mL) stored in a sample chamber flows through the membrane under vacuum pressure and then is collected in a waste chamber within 2 min. Then, with valve control, washing fluids (three different concentrations of ethanol) were sequentially connected to the silica membrane and are collected in the waste chamber. During the serial washing process, proteins and other substance on the silica membrane would be washed out for 3 min. In the drying step, room-temperature air is sucked in and flown through the silica membrane of the spin column for 5 min. During the drying step, all residues of ethanol are completely evaporated and removed. In the elution step, the elution buffer is loaded on the silica membrane and a mineral oil is stacked on the elution buffer. Then, by connecting vacuum pump, the stacked fluids flow through the membrane and collected in the collection tube for 1 min.

The entire process of cfDNA extraction using the PIBEX chip can be completed within 15 min, since each step is sequentially processed. All extraction steps can be controlled using combinations of different modes of two rotating microfluidic valves. For example, in the DNA binding step, the flow channels of the PIBEX chip are connected from the plasma sample chamber to waste (Fig. 1c). A photograph of the microfluidic PIBEX chip is shown in Fig. 1d. The chip, which measures 14 mm in width, 100 mm in length, and 40 mm in height, consists of chambers, valves, and microfluidic circuits. The chip contains six buffer chambers that accommodate the plasma sample, three washing buffers, elution buffer, and mineral oil, and two rotating microfluidic valves (a six-way selection valve and a switch valve) (Fig. S1). It is operated using a mini vacuum pump. As shown in Fig. 1d, the final elution tube can be directly used for a thermal cyclic PCR process. Thus, the current design of the PIBEX chip and the system can be further developed as a sample-to-answer system.

The PIBEX is a highly integrated system chip with microfluidics even though it is not apparently shown. The main operating principle of the PIBEX is based on the microfluidics associated with surface tension in silica membrane pores, which was described in our previous study.^36^ In the microscale membrane pores, the surface tension is the major hindrance to deteriorates recovery rate of elution buffer. The main advantage of PIBEX is to resolve the surface tension in microscale by stacking immiscible oil on the top of elution buffer effectively.^36^ Owing to the microfluidic-based PIBEX chip, the entire extracting process consisting multiple discrete steps was replaced with one continuously operating process within a chip, which enables to eliminate risk of cross contamination, reduction of work flow consistency and requirement of skilled operators. The whole system chip consisting of chambers and the silica membrane was connected with microchannels (600 μm in width and 400 μm in height) and micro-valves. The channel dimensions are also carefully designed to minimize any loss of buffers by absorption on fluidics walls. Thus, the high density integrated microfluidic chip, PIBEX providing a continuous process of cfDNA extraction in a chip for several steps, enables to eliminate risk of cross contamination, reduction of work flow consistency and requirement of skilled operators.

### Microfluidic characteristics for cfDNA extraction

**Fig. 2:**
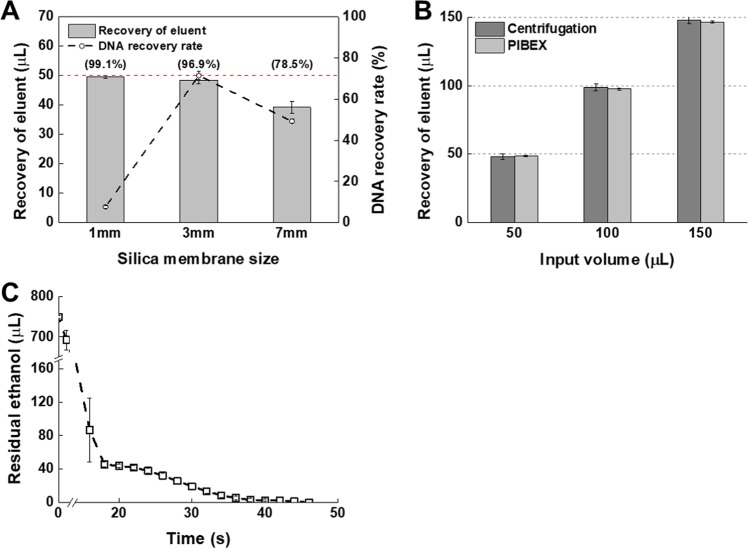
Characteristic analysis of a microfluidic PIBEX chip. Eluting, pushing with oil, and drying steps were optimized for the PIBEX application in a microfluidic chip. **a** Membrane size effect on recovery volume of eluent (bar chart) and DNA recovery rate (dotted line) in the PIBEX method were investigated (*n* = 3). The target initial volume of eluent was 50 μL. The numbers in parentheses denote the percentage of eluent recovery. **b** Recovery amount of eluent were investigated in centrifugation, vacuum only, and PIBEX chip methods (*n* = 3). Initial eluent volume varies from 50–150 μL. **c** Residual ethanol remaining inside silica membrane was monitored in pressure-driven air flow (*n* = 3). The error bars indicate standard deviation.

Since precision cfDNA extraction is a prerequisite for precision liquid biopsy, performance analysis of precision cfDNA extraction is important. Two major indices are used to gauge cfDNA extraction performance: recovery of the eluent and the rate of DNA recovery. Preliminary experiments revealed that the size of the silica membrane is a major influential factor. Thus, the present experiments were conducted with three different diameters of silicon membranes (1, 3, and 7 mm), as shown in Fig. 2a. To measure the DNA recovery rate, the 180 bp lambda DNA PCR product was spiked into the plasma of a healthy control. For comparison, the volumes of eluent and the plasma were fixed at 50 μL and 1 mL, respectively.

When considering both indices, 3 mm silicon membranes had the best performance, with a recovery rate of 96.9% for the eluent and 78% for the DNA. The small (1 mm) membrane produced a very high eluent recovery rate of 99.1%, but extremely poor recovery of DNA (5%). The difference in the extraction performance may be due to the surface area of silica membrane to capture cfDNAs. Considering the identical height and porosity of silica membranes, the DNA capture capacity is proportional to the surface area of the membrane. Therefore, a higher yield in larger silica membranes is natural. For the 7 mm-diameter membranes, the eluent recovery rate decreased to 78.5%, which induced a low recovery rate of DNA (50%). The results may not be generalized for wide ranges of sample volumes (0.5 to approximately 5.0 mL). Of note, that the size of the silica membrane in the QIAamp Circulating Nucleic Acid Kit (QIAGEN, USA) is 7 mm, and 5 mL of plasma can be applied to it. Therefore, the 3 mm-diameter membrane was selected for the 1 mL plasma capacity in the PIBEX chip. For the convenience of the user, the amount of elution buffer was varied from 50 μL to 150 μL to adjust the final cfDNA concentration. The recovery of the final eluent in the PIBEX chip was comparable to centrifugation (Fig. 2b).

Since centrifugation-based ethanol drying is replaced by air flow drying in the PIBEX method, ethanol removal was carefully examined. Complete removal of ethanol is critical, since even a small amount of residual ethanol can inhibit DNA amplification. In the PIBEX chip, ethanol buffer was allowed to flow through the membrane followed by air at room temperature. The weight of residual ethanol in the silica membrane was monitored during air flow using a precision mass balance. The residual ethanol quickly dried for the first 18 s and then slowly dried thereafter (Fig. 2c). With air flow, complete drying was achieved within 47 s. A drying time of 3 min was more than sufficient for stable sample preparation for the PIBEX chip. Subsequent experiments demonstrated that PCR amplification was not inhibited, confirming the complete removal of ethanol by the air-drying process.

### Performance analysis of cfDNA extraction using the PIBEX microfluidic chip

**Fig. 3:**
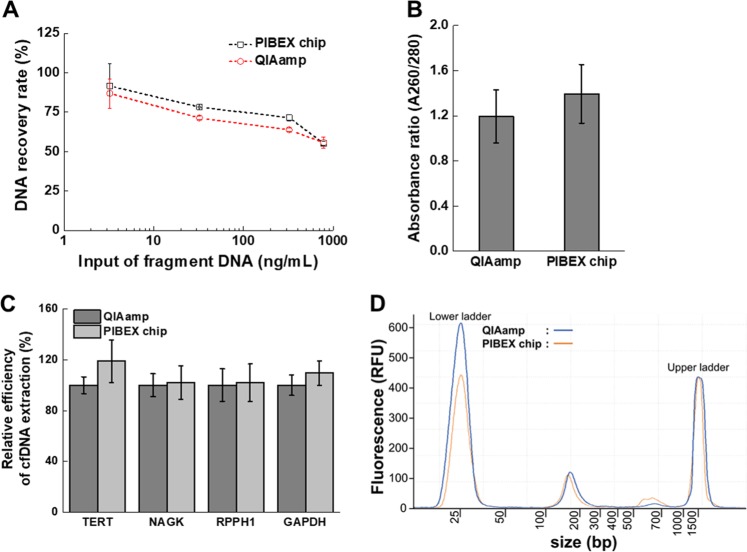
Performance analysis of cfDNA extraction using a microfluidic PIBEX chip. The performance of cfDNA extraction in the PIBEX chip compared to the commercial product QIAamp. **a** DNA recovery rates according to input of fragment DNA was investigated in three experiments. **b** Purity of final eluents was compared by absorbance ratio at A260/280 (*n* = 5). **c** Relative efficiency of cfDNA extraction in healthy plasma sample was measured by real-time PCR with the four reference genes: *TERT*, *NAGK*, *RPPH1*, and *GAPDH*. The relative efficiency was calculated based on cfDNA extraction in QIAamp (*n* = 8). **d** Size analysis of extracted DNA from human plasma of healthy control was measured by microelectrophoresis. The error bars indicate standard deviation.

The performance of the PIBEX chip was analyzed in more detail compared to the QIAamp Circulating Nucleic Acid Kit. For comparison, the 180 bp PCR product amplified from lambda DNA was spiked into human plasma of a healthy control. The results of the comparison of the two extraction methods are shown in Fig. 3. No significant differences were evident between the QIAamp kit and PIBEX chip in terms of DNA recovery rate (Fig. 3a) and purity (Fig. 3b). The PIBEX chip produced a somewhat higher DNA recovery rate than QIAamp without statistical significance. The DNA recovery rate of both methods decreased with increasing amount of DNA. For the highest range of input DNA (800 ng/mL), the recovery rate was reduced by 55% because capacity of silica membrane to absorb DNA was exceeded. In the control experiment using spiked lambda DNA, there is original cfDNA in the plasma sample as well so that DNA recovery may be affected by the membrane load. The amount of DNA actually present in plasma can be up to double in the range of tens of ng/mL, which is the normal range of human cfDNA amount.^10,11^ Fortunately, the DNA recovery rate reaches 70 to 80% at a concentration of 10 to 100 ng/mL of input DNA (Fig. 3a). Even considering the presence of the original cfDNA, the ability to extract cfDNA from PIBEX and QIAamp can be interpreted as about 70–80%, since the recovery of input lambda DNA at 200 ng/mL was maintained as about 70% at twice the normal range. The QIAamp blood DNA Midi Kit has demonstrated a DNA extraction efficiency of 18.6 to 38.7%.^42^ The extraction efficiency was improved in the Circulating Nucleic Acid Extraction Kit but was still only approximately 70% in human serum.^37^

After the initial feasibility tests, we extracted cfDNA from the plasma of healthy controls and used real-time PCR to ensure that the present technique is sufficient for real human cfDNA extraction as quantifying specific reference genes— telomerase reverse transcriptase (*TERT*), N-acetyl-D-glucosamine kinase (*NAGK*), ribonuclease P RNA component H1 (*RPPH1*), and glyceraldehyde-3-phosphate dehydrogenase (*GAPDH*) (Fig. 3c). These genes are constitutively expressed (housekeeping) genes that maintain basic cellular functions and are expressed in all cells under normal and pathophysiological conditions. The comparative extraction efficiency between QIAamp and PIBEX chip was investigated by Ct value calculation (Fig. S2). In addition, the levels of the four genes in cfDNA extracted by either QIAamp or PIBEX chip were compared (Fig. S3). PIBEX displayed slightly higher (but not statistically significant) extraction efficiencies of *TERT* and *GAPDH*, compared to QIAamp (Fig. 3c).

The quality of the extracted DNAs from a healthy control was examined using microelectrophoresis (Fig. 3d). A cfDNA peak with an average size of 180 bp was clearly evident between the lower and upper ladders. There was no contamination associated with genomic DNA (gDNA), which would have appeared in the large size range. The sample must not be contaminated with gDNA contamination, since this would produce inaccurate liquid biopsy findings. To avoid gDNA contamination, plasma was immediately isolated from leukocytes and other blood cells after blood sampling. There was no significant difference in the amplitudes of peaks indicative of the quantities of cfDNA between the two methods.

### Comparative validation with synthetic plasma and ddPCR analysis

To confirm the clinical potential of the PIBEX chip for liquid biopsy, we used the Multiplex I cfDNA Reference Standard Set (Horizon Discovery, UK), which contains eight cancer-relevant mutations, including B-Raf proto-oncogene, serine/threonine kinase (*BRAF*), epidermal growth factor receptor (*EGFR*), Ki-ras2 Kirsten rat sarcoma viral oncogene homolog (*KRAS*), neuroblastoma RAS Viral (V-Ras) Oncogene Homolog (*NRAS*), and *PIK3CA*, at 5%, 1%, and 0.1% allelic frequencies in synthetic plasma. These mutations are associated with multiple forms of cancer including breast, colorectal, pancreatic, and lung. Detailed results of quality test of the cfDNA reference set in synthetic plasma are shown in Fig. S4. Using the reference standard set, we extracted DNA with the PIBEX chip and QIAamp, then analyzed ctDNAs by ddPCR. Each ddPCR test required 500 μL of cfDNA reference sample.

The comparative ddPCR was analyzed for four target ctDNAs between QIAamp and PIBEX. The target DNAs were *PIK3CA* E545K, *EGFR* L858R, *KRAS* G12D, and *NRAS* Q61K. They co-exist with four other mutations in synthetic plasma. Since these mutations occur frequently in primary cancers, they are potential candidates for a liquid biopsy assay based on the number of variant calls.^43^ The standard reference set consisted of four different fractions (0%, 0.1%, 1%, and 5%) containing eight different variations, including the aforementioned mutants. However, the actual allelic frequencies of each reference cfDNA slightly varied from 0% to 6.3% according to target variants (Table S4).

**Fig. 4:**
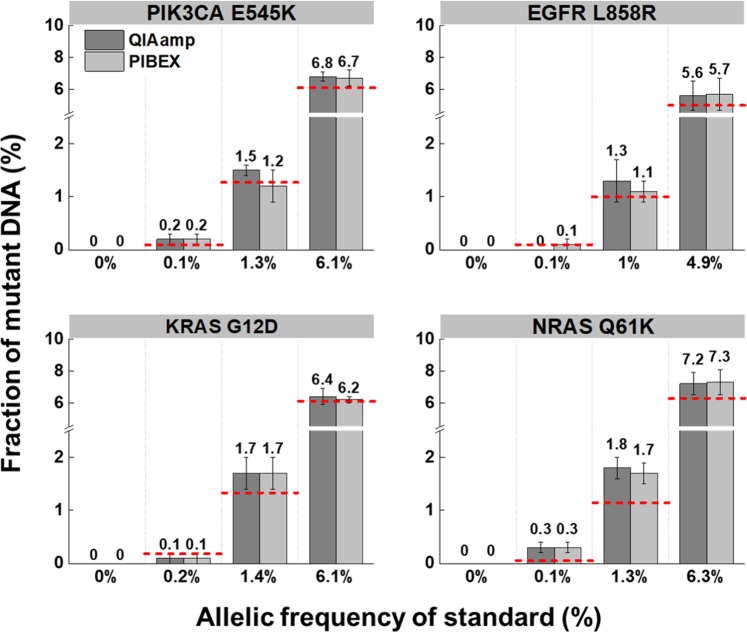
Comparison analysis of ctDNA extraction between PIBEX chip and QIAamp using ddPCR. Standard ctDNA sample in synthetic plasma were extracted by QIAamp and PIBEX chip. Four mutations (*PIK3CA* E545K, *EGFR* L858R, *KRAS* G12D, and *NRAS* Q61K) were measured by ddPCR. Prepared allelic frequency of standard ctDNA samples varied from 0–6.3% according to target variants. Red lines represent the expected fraction of mutant DNA for each experiment. The error bars indicate standard deviation (*n* = 4).

The ddPCR analyses successfully detected the mutations in all prepared allelic frequencies ranging from 0.1% to 6.3%. For example, the four standard sets contained *PIK3CA* E545K mutants at 0%, 0.1%, 1.3%, and 6.1%. QIAamp extracted 0%, 0.2%, 1.5%, and 6.8% of the *PIK2CA* E545K mutants, respectively, while PIBEX, respectively, extracted 0%, 0.2%, 1.2%, and 6.7% (Fig. 4). There were no significant differences in mutant fractions in ctDNA extraction between QIAamp and the PIBEX chip. Similar results of equivalent performance were obtained for the other three mutations between QIAamp and PIBEX. Thus, both methods successfully extracted specific target DNAs at a concentration as low as 0.1%.

### Clinical validation in metastatic breast cancer with next generation sequencing (NGS) and ddPCR analysis

**Fig. 5:**
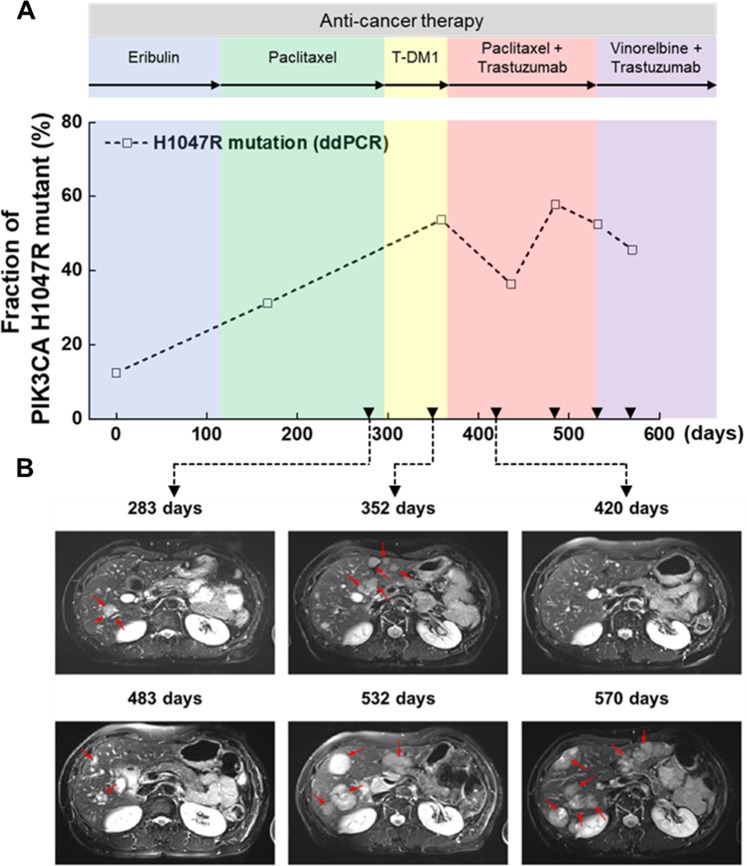
Clinical validation of PIBEX chip in liquid biopsy of breast cancer. The *PIK3CA* mutation in *HER-2* type of breast cancer was monitored in liver metastasis using DNA extracted from the PIBEX chip. **a** Fraction change of *PIK3CA* H1047R mutation in ctDNA was detected by ddPCR. Each cancer therapy image is labeled with the background color of the graph. **b** Hepatic metastasis from breast cancer in abdominal MRI images. Red arrows indicate metastasis of breast cancer in liver. Inverted triangles of **a** represent the matching MRI images of **b**.

To examine the clinical utility of the PIBEX chips, clinical samples were applied to the PIBEX chip to extract cfDNA, which was analyzed by ddPCR. The clinical samples were serially obtained from a 58-year-old female patient diagnosed with *HER-2* type breast cancer for 570 days. The patient showed a high fraction (9.15%) of pathogenic mutation *PIK3CA* H1047R at baseline by NGS analysis (Table S5). Therefore, *PIK3CA* H1047R was selected as a target mutant in the ddPCR assay. Cancer treatments and medical imaging were conducted on serial samples. The fraction of target DNA linearly increased for 365 days (Fig. 5a). However, serial chest computed tomography (CT) images showed a decrease at the lymph node in the same time period (−7 to 276 days) (Fig. S5a). The findings indicated the metastasis of the cancer to another organs.

Initial metastasis to the liver was found at day 283 using abdominal magnetic resonance imaging (Fig. 5b). The liver had been clear in abdominal CT images until day 276 (Fig. S5b). Aggravation of liver metastasis was observed at day 352 and the mutant fraction of *PIK3CA* H1047R reached 53.7%. Temporary decrease of tumor lesions in liver and reduction of *PIK3CA* mutant fraction to 36.4% were observed after the combination treatment with paclitaxel and trastuzumab at day 420. However, the hepatic tumor recurred, and the mutant fraction increased to 57.9% at day 483. After the secondary peak, the *PIK3CA* mutant fraction decreased but still remained high (>45%). Overall tumorigenesis in the liver was observed in MRI images after 532 days. Data at days 420 and 570 were double-checked with NGS data; a good correlation with the ddPCR results was evident. Except for the *PIK3CA* mutation, no other significant mutation was found (Table S5).

## Discussion

Accumulating evidence supports the existence of tumor DNA circulating in the blood stream, which can be detected in small sample volumes using state-of-the-art technology.^38,44,45^ The advantage of liquid biopsy in terms of its ease of sampling of blood compared to tissue has attracted research and clinical attention for the longitudinal monitoring of cancer and response to treatments.^45^ Since cfDNAs in blood are present at very low concentrations and ctDNA exist at highly rare frequency, extraction followed by precision detection is necessary. Since the extraction process is preceded by downstream detection or sequencing, the extraction yield may strongly affect the precision of downstream processes. Depending on materials, geometry, and the adopted method, extraction results can vary considerably.^37,38,44,46,47^ Among the commercial products, QIAamp and Maxwell RSC (Promega, USA) have shown reliable extraction results for downstream PCR analyses.^44^ However, QIAamp is preferred for NGS, with other extraction techniques posing technical difficulties for the preparation of NGS libraries. Considering DNA extracting material, silica has stable and high yield regardless of application type such as membrane^47^ or micro-bead.^37^ In recent studies, it has been reported that a large surface area using micro-pillars can effectively improve the cfDNA extraction efficiency of liquid biopsies even with surface other than silica (surface-confined carboxylic acid functionalities on PC with UV/O_3_ treatment^46^ and amine-modification with DTBP^38^). The PIBEX chip adopting silica membrane showed the similar performance as that of QIAamp. These performance results can be evaluated as successful since they are obtained with shortened operation time and increased ease of operation. These results are summarized in Table 1.

**Table 1:** Summary of cfDNA extraction.

Category	Surface material	Description	Recovery rate	Sample	Mutant	Mutant fraction	Author (ref.)	Year
Pillars	PC, COC	UV/O_3_ treatment	92%	cfDNA	*KRAS* G12V, *KRAS* G12D, *KRAS* G13S	0.1–10%	Campos et al. ^46^	2018
Membrane	Silica	PIBEX	80%	cfDNA	*PIK3CA* E545K, *PIK3CA* H1047R, *EGFR* L858R, *KRAS* G12D, *NRAS* Q61K	0.1–57.9%	This study	–
Membrane	Silica	QIAamp circulating nucleic acid kit (QIAGEN)	80%	cfDNA	*BRAF* V600E	–	Diefenbach et al. ^47^	2018
Magnetic bead	Cellulose	Maxwell RSC ccfDNA plasma kit (Promega)	60%					
Magnetic bead	Unknown	MagMax cell-free DNA isolation kit (Applied Biosystems)	50%					
Bead	Silica	Rotating disc	75%	cfDNA	*EGFR* L858R, *EGFR* T790M	2.5–80%	Kim et al. ^37^	2018
Micro channel	Amine-modified firm	Adding DTBP	–	cfDNA	*BRAF* V600E, *KRAS* G12D, *KRAS* G13D	0.5–80%	Jin et al. ^38^	2018

*PC* polycarbonate, *COC* cyclic olefin copolymer, *DTBP* dimethyl dithiobispropionimidate

The loading volume of plasma was carefully determined with considering clinical needs for practical use. From a standpoint of analyst, as much as large amount of plasma sample is preferred for cfDNA analysis due to inherent low amount and rare fraction of ctDNA in blood flow in early stage of disease. However, it is not easy to collect large amount of blood samples for elderly or weakened cancer patients. Frequent sampling of large amount of blood is more so. Furthermore, such samples must also be used for various tests, so they cannot be used as a test at a time. Considering the clinical conditions, therefore, we carefully determined the required plasma capacity to 0.5–1 mL of plasma (i.e., 1–2 mL of whole blood sample) for each test to extract cfDNA in PIBEX chip. This study has made ctDNA monitoring in the case of advanced breast cancer, but the possibility of using a small sample volume in early clinical disease needs to be investigated through further clinical studies.

One can raise a fundamental question on liquid biopsy how a small amount of sample can be representative of all DNA mutations in the circulating blood stream. For instance, the first sampling rate for liquid biopsy is 0.1% (5 mL out of 5 L). During sample preparation and sensing processes, DNA recovery rate is about 80% and sampling rate for PCR is about 5% from eluent liquid (100 µL) to PCR sample (~5 µL). Despite these limited sampling rates and recovery rates, ctDNA in the body has been detected at low concentrations.^46^ The results imply that DNA exist uniformly and even the concentration of ctDNA in µl-scale sample aliquot would be the same as that in ml-scale sample volume. It is reasonable to assume uniform distribution of nanoscale substances such as DNA in uL-scale volume (or mm^3^). It is worthy to note that cfDNA is highly fragmented with a short peak fragment of approximately 180 bp with an approximate length of 61.2 nm, assuming that 1 bp of DNA is 0.34 nm.

The cfDNA extraction chip described here emphasizes the need for sample preparation techniques that can be easily utilized in liquid biopsies obtained during cancer treatment. The previously introduced PIBEX method has been successfully implemented in a microfluidic chip consisting of elaborate microfluidic circuits and valves, a simple and rapid ethanol drying process, and an immiscible liquid-pushing process. The PIBEX chip can be used to quickly and simply extract cfDNA within 15 min by replacing centrifugation with vacuum pressure during the entire extraction process. Systematic testing revealed the equivalent performance of the PIBEX chip to that of conventional QIAamp spin column. The longitudinal monitoring of a breast cancer patient was usefully conducted in this study. However, further in vitro and clinical studies with appropriately powered sample size are required to further evaluate the performance and clinical value of monitoring using the PIBEX chip.

Conventional automation systems for nucleic acid extraction have been limited to the application of an internal centrifuge or liquid handler to replace the manual process. Beyond existing development trends, the use of disposable PIBEX chips has revolutionized operating time and convenience. The development of a fully automated operating system for the current PIBEX chip is expected to pave the way for more innovations in cfDNA extraction. The PIBEX chip can be applied to a wide range of targets (genomic DNA, plasmid DNA, RNA, and viral nucleic acids) and samples (plasma, serum, urine, or cell culture media) by adjusting the selection of appropriate reagents with varying ionic strength^46^ and chaotropic salt.^37,48^ Thus, this study demonstrates the potential use of PIBEX chips in clinical, as well as research environments. In addition, the PIBEX chip is expected to further facilitate the application of a final sample-to-answer system in liquid biopsy that can address unmet clinical needs in cancer management.

## Methods

### Blood sample preparation

This study was conducted according to the principles of the Declaration of Helsinki. The Institutional Review Board of Korea University Anam Hospital, Seoul, Republic of Korea, approved this study protocol (IRB project number: 2016AN0090). All participants provided written consent for the samples. Blood samples were collected in 3 mL K2-EDTA vacutainers (Becton Dickinson, USA) from the median cubital vein. Detailed information of the samples is provided in Table S1. Plasma was isolated after centrifuging whole blood at 1900 × g for 10 min using a model 1248 apparatus (LABOGEN, Denmark) followed by 12,000 × g for 15 min. The entire process of plasma separation was carried out within 2 h after blood collection.

### Standard cfDNA sample preparation

Two types of standard samples were used: cfDNA standard sample and cfDNA reference set in synthetic plasma. The cfDNA standard sample was obtained by spiking 180 bp PCR product from lambda DNA in human plasma collected from healthy controls. The lambda DNA was purchased from Bioneer (South Korea). The primer set (forward primer: 5-CAGCGATGGATTTTATTCTGG-3 and reverse primer: 5-CGTTATCCGTATCCTGAGC-3) was synthesized by GENOTECH (South Korea). The amplification was performed under the following conditions: polymerase activation at 95 °C for 20 s, 40 cycles of denaturation at 95 °C for 1 s, and 40 cycles of annealing and extension at 50 °C for 20 s. The PCR product was purified using the QIAquick PCR Purification Kit (QIAGEN). The purified fragmented DNA (135 ng) from lambda DNA was spiked in 500 μL of healthy human plasma. The detailed information of the primer set is provided in Table S2. The cfDNA reference set in synthetic plasma (HD780) was purchased from Horizon Discovery. The reference set consisted of four different fractions (0%, 0.1%, 1%, and 5%) containing eight different variations (*EGFR* L858R, *EGFR* ΔE746-A750, *EGFR* T790M, *EGFR* V769-D770insASV, *KRAS* G12D, *NRAS* Q61K, *NRAS* A59T, and *PIK3CA* E545K). The detailed information of the cfDNA reference set in synthetic plasma is provided in Table S4.

### Fabrication of PIBEX chips

The PIBEX chip consisted of five parts: top plate, body plate, silicone gasket, two jigs, and two microfluidic valves (6-way selection valve and switch valve). Each part of the PIBEX chip was manufactured using a TinyCNC-SC milling machine (Tinyrobo, South Korea). The horizontal fluid channels were 800 μm in width and 500 μm in height, and the vertical fluid channels were 1 mm in diameter. The body and top plates were bonded using MC100 acrylic bond (HANDEUL, South Korea). The fluid channels positioned on the bottom side of the body plate were sealed with MicroAmp™ 48-Well Optical Adhesive Film (Applied Biosystems, USA). Each chamber was designed to accommodate the required volume of reagents as follows: 4.8 mL (sample chamber), 900 μL (washing buffer chamber 1–3), 450 μL (elution and mineral oil chamber), 12 mL (waste), and 660 μL (collection chamber). The silicone gasket was made of a condensation-type silicone (Moldmaster pop; Molkang, Korea) using a silicone gasket mold manufactured by a precision CNC machine. To fabricate the silicone gasket, liquid silicone and a curing agent in the condensation-type of silicone were mixed at a 100:1 ratio, then completely loaded in the silicone mold. After curing at room temperature for 12 h, the silicone gasket was carefully detached. In the middle of the gasket, a stainless-steel tube (ISM584; IDEX Health and Science, USA) with a 0.84 mm inner diameter, 1.27 mm outer diameter, and 11.5 mm length was inserted as a guide line to the center of the silica membrane. A commercial rubber stopper (Rheomeditech, South Korea) was used as the lid of the collection chamber and waste. To apply vacuum pressure to the PIBEX chip, a vacuum pump (SC5002PM; Skoocom Electronic, China) was connected to the vacuum connection site in the chip using a conical adapter (P794; IDEX Health and Science, USA) and tubing (SC0374T, IDEX Health and Science).

### Spin column-based cfDNA extraction

The spin column-based cfDNA extraction was performed following the recommended protocol. The reagents in the QIAamp Circulating Nucleic Acid Kit (55114; QIAamp) were used to isolate cfDNA. Lysate buffer (ACL) containing 1 μg of carrier RNA was prepared prior to the experiment. The volume of human plasma varied from 500 μL to 1 mL. Proteinase K solution at 1/10 of the required plasma volume, required human plasma, and ACL at 4/5 of the required plasma volume were sequentially added to a 15 mL conical tube. The mixture was homogeneously mixed by vortexing for 30 s and incubated at 60 °C for 30 min. Binding buffer (ACB) at 9/5 of required plasma volume was added to the mixture. After vortexing for 30 s, the final mixture was incubated on ice for 5 min. The volume of final mixture varied from 1.85 to 3.70 mL. A spin column with a provided extender was mounted on the QIAvac 24 Plus manifold (QIAGEN), which was connected to a vacuum pump. In the cfDNA binding step, the final mixture was added to the spin column and the vacuum pump was turned on until the final mixture completely passed through the silica membrane. After the cfDNA binding procedure, the extender was removed. In the washing step, 600 μL of washing buffer 1 (ACW1), 750 μL of washing buffer 2 (ACW2), and 750 μL of 99% ethanol as washing buffer 3 were sequentially passed through the silica membrane. In the drying step, the spin column in a 2 mL collection tube was centrifuged at 12,000 × g for 3 min, then the spin column was transferred to a fresh 1.5 mL elution tube. Finally, in the elution step, 50 μL of elution buffer was carefully applied to the center of the spin column and centrifuged at 12,000 × g for 1 min.

### cfDNA extraction using the PIBEX chip

In the PIBEX chip experiment, the reagents from the QIAamp Circulating Nucleic Acid Kit (55114; QIAamp) were used and 500 μL of plasma samples from a healthy control and cancer patient were applied. All reagents were pre-loaded into each reagent chamber: 600 μL of ACW1, 750 μL of ACW2, 750 μL of ethanol, 50 μL of elution buffer, and 100 μL of mineral oil (330760; Sigma-Aldrich, USA). After loading 1.85 mL of the final mixture of cfDNA, the vacuum pump was turned on until the end of the entire process. All steps of cfDNA extraction on a PIBEX chip were performed under a vacuum pressure at 200 kPa. Microfluidic valves were either manually rotated by a user using a flat-head screwdriver or by a rotating motor. In the DNA binding and washing steps, the reagent chambers (sample, ACW1, ACW2, and 99% ethanol) were sequentially connected to the silica membrane with a rotation of 60° of the 6-way valve, and the switch valve was positioned to connect to the silica membrane and waste. After the washing step, the silica membrane was dried completely by air flow for 3 min. In the elution and oil-dry steps, the switch valve was rotated by 180° to connect the silica membrane to the collection chamber, and the 6-way valve was sequentially positioned in the elution buffer chamber and mineral oil chamber. The silica membrane was replaced in every experiment to prevent cross contamination. Microfluidic valves and channels were thoroughly washed with 5 mL of 99% ethanol and were carefully dried with a flow of nitrogen gas. After 10 min of additional drying at room temperature, the PIBEX chip was reused. To determine whether there was cross contamination, the PIBEX experiment was performed using phosphate buffered saline instead of plasma. The lack of residual DNA confirmed that there was no cross contamination.

### Calculation of DNA extraction efficiency

DNA extraction efficiency was calculated based on specific DNA recovery by comparing the absolute copy number of DNA detected in qPCR to the theoretically expected value assuming 100% DNA extraction efficiency:

DNA extraction efficiency (%) = 100 × (DNA copies detected/DNA copies expected)

### Quantification of cfDNA in eluents by qPCR

To measure the amount of cfDNA in eluents, real-time PCR assays were performed with a StepOne™ Real-Time PCR system (Applied Biosystems) using the *TERT*, *NAGK*, *RPPH1*, and *GAPDH* as reference (Table S3). Real-time PCR analysis was performed in triplicate on a sample obtained from a healthy control using TaqMan Fast Advanced Master Mix (Thermo Fisher Scientific, USA). A standard curve of each reference gene was measured in seven points of 5-fold dilutions using human genomic DNA (Promega). The primer and probe sets were synthesized from GENOTECH (South Korea); detailed information is presented in Table S3. In the experiment, two-step thermocycling consisted of polymerase activation at 95 °C for 20 s, 40 cycles of denaturation at 95 °C for 1 s, 40 cycles of annealing, and extension at 55 °C for 20 s.

### Purity, quantity, and quality check of extracted cfDNA

Purity of cfDNA was determined by measuring the ratio of the absorbance at 260 nm and 280 nm of 1 μL of final eluent with a DS-11 FX + spectrophotometer (Denovix, USA). Elution buffer was used as the negative control to blank the spectrophotometer and remove background noise. DNA concentration was measured using a spectrometer with the Quant-iT PicoGreen dsDNA Assay Kit (Thermo Fisher Scientific). The fluorescence intensity of PicoGreen was measured to determine the DNA recovery rate in the final eluent. The recovery rate was calculated by standardizing with a known amount of spiked lambda DNA fragments with subtraction of the inherent cfDNA amount in the donor. To check the quality of the extracted DNA, we used the Tapestation 4200 automated instrument for microelectrophoresis (Agilent, USA) with High Sensitivity D1000 tape (Agilent).

### Measurement of ctDNA fraction in eluents by ddPCR

The primer sets for ddPCR for *KRAS* G12D, *NRAS* Q61K, *PIK3CA* E545K, and *EGFR* L858R were purchased from Bio-Rad (USA). The tests for each primer set were performed in triplicate. The 20 μL of reaction solution was made with the following recipe: 10 μL of Supermix (ddPCR Supermix for Probes; Bio-Rad), 1 μL of each primer set, 4 μL of final cfDNA eluent, and 4 μL of distilled water (Thermo Fisher Scientific). The PCR reaction was performed according to the recommended protocol; polymerase activation at 95 °C for 10 min, 40 cycles of denaturation at 94 °C for 30 s, 40 cycles of annealing/extension at 60 °C for 1 min, and enzyme deactivation at 98 °C for 10 min. The produced was stored at 4 °C.

### Measurement of ctDNA fraction in eluents by NGS

The cfDNA for NGS analysis was extracted using the QIAamp Circulating Nucleic Acid Kit (55114, QIAamp, USA) from 2 mL of plasma sample. Libraries were prepared with the SureSelect XT low input protocol (Agilent Technologies, USA) using the Axen Cancer Panel 1 (88 genes, Table S6) developed by Macrogen (Macrogen, Seoul, South Korea). The libraries of each sample were individually indexed and molecularly barcoded. To ensure the product size of 200 to 400 bp, a quality check was conducted using the model 2100 Bioanalyzer (Agilent Technologies). The libraries were quantitated using the Qubit dsDNA HS Assay Kit and Qubit 2.0 fluorometer (Life Technologies, USA). The libraries were sequenced to a depth in the range of about 5000× with paired-ends (2 × 150 bp) on a NextSeq500 (Illumina, USA).

### Statistical analysis

The statistical analysis performed for quantification of results is indicated in the figure legends. All quantitative results are expressed as mean values ± standard deviations. Experimental results were independently performed at least three times, unless stated otherwise in the figure legends.

## Supplementary information

Supplementary information

## Data Availability

ctDNA next-generation sequencing data are publicly available in the NCBI Sequence Read Archive (SRA) under the accession number PRJNA605079. The other data sets generated during and/or analyzed during this study are available from the corresponding author on reasonable request.
