# Supplementary material for: Precision cell-free DNA extraction for liquid biopsy by integrated microfluidics
Source: NPJ Precis Oncol. 2020 Feb 24;4:3. doi: 10.1038/s41698-019-0107-0 (PMC7039987; doi:10.1038/s41698-019-0107-0)
Supplement: Supplementary file 1 — Supplementary information [file 41698_2019_107_MOESM1_ESM.pdf]

Supplementary information

Centrifugation-free cfDNA extraction in a microfluidic chip using immiscible reagent and vacuum for liquid biopsy of cancer

Hoyoon Lee[1], Chanhee Park[1], Wonhwi Na[2], Kyong Hwa Park[3] and Sehyun Shin[1]

[1]School of Mechanical Engineering, Korea University, Seoul 02841, Republic of Korea

[2]Department of Micro/Nano Systems, Korea University, Seoul 02841, Republic of Korea

[3]Division of Oncology/Hematology, Department of Internal Medicine, Korea University College of Medicine, Seoul 02841, Republic of Korea

Correspondence: Sehyun Shin (lexerdshin@korea.ac.kr)

Hoyoon Lee and Chanhee Park contributed equally to this work.

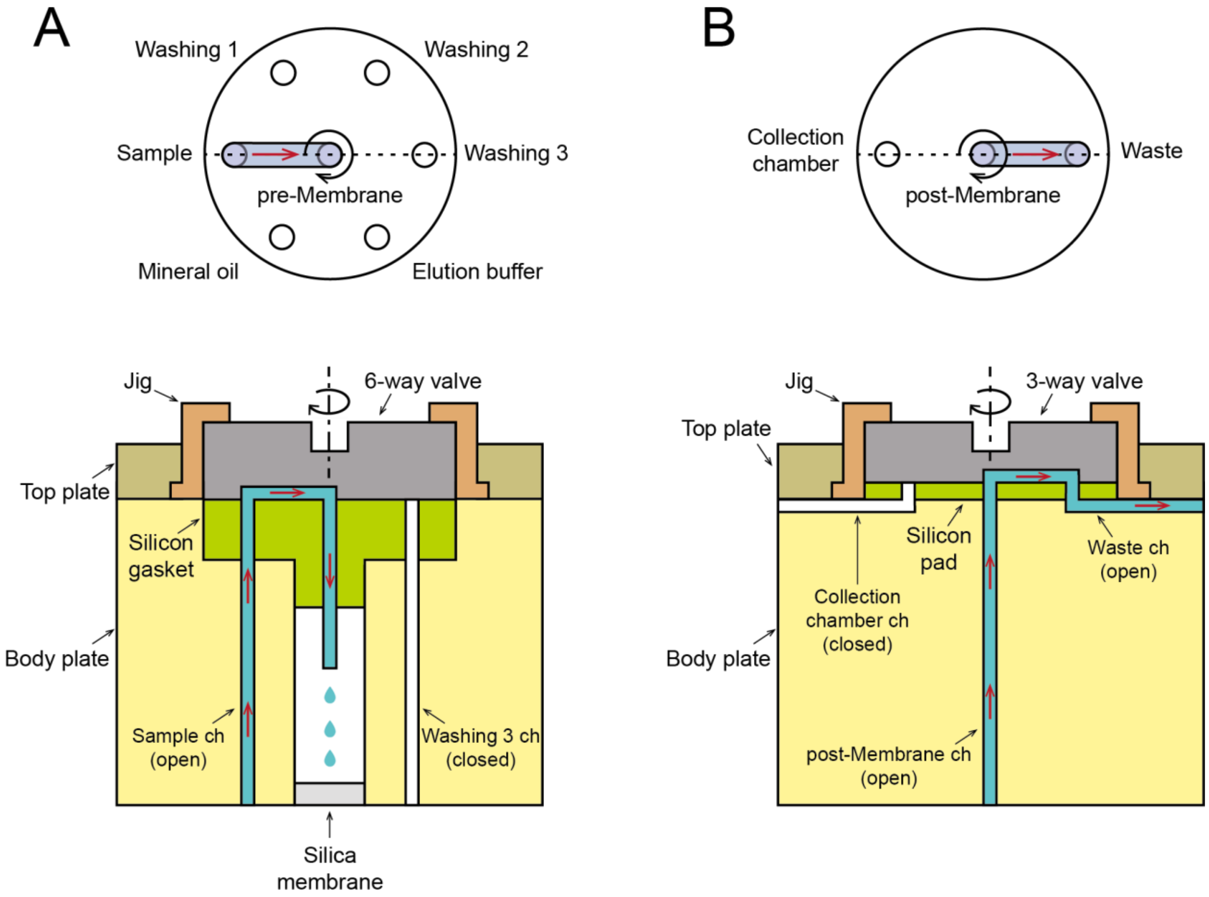

Fig. S1 Schematic images of microfluidic valves in the PIBEX chip. A) Top view shows a schematic image for the microfluidic 6-way selection valve (top) and section view at dotted line in top view (bottom). B) Top view shows a schematic image for the microfluidic switch valve (top) and section view at dotted line in top view (bottom). Red arrows indicate flow direction in a channel.

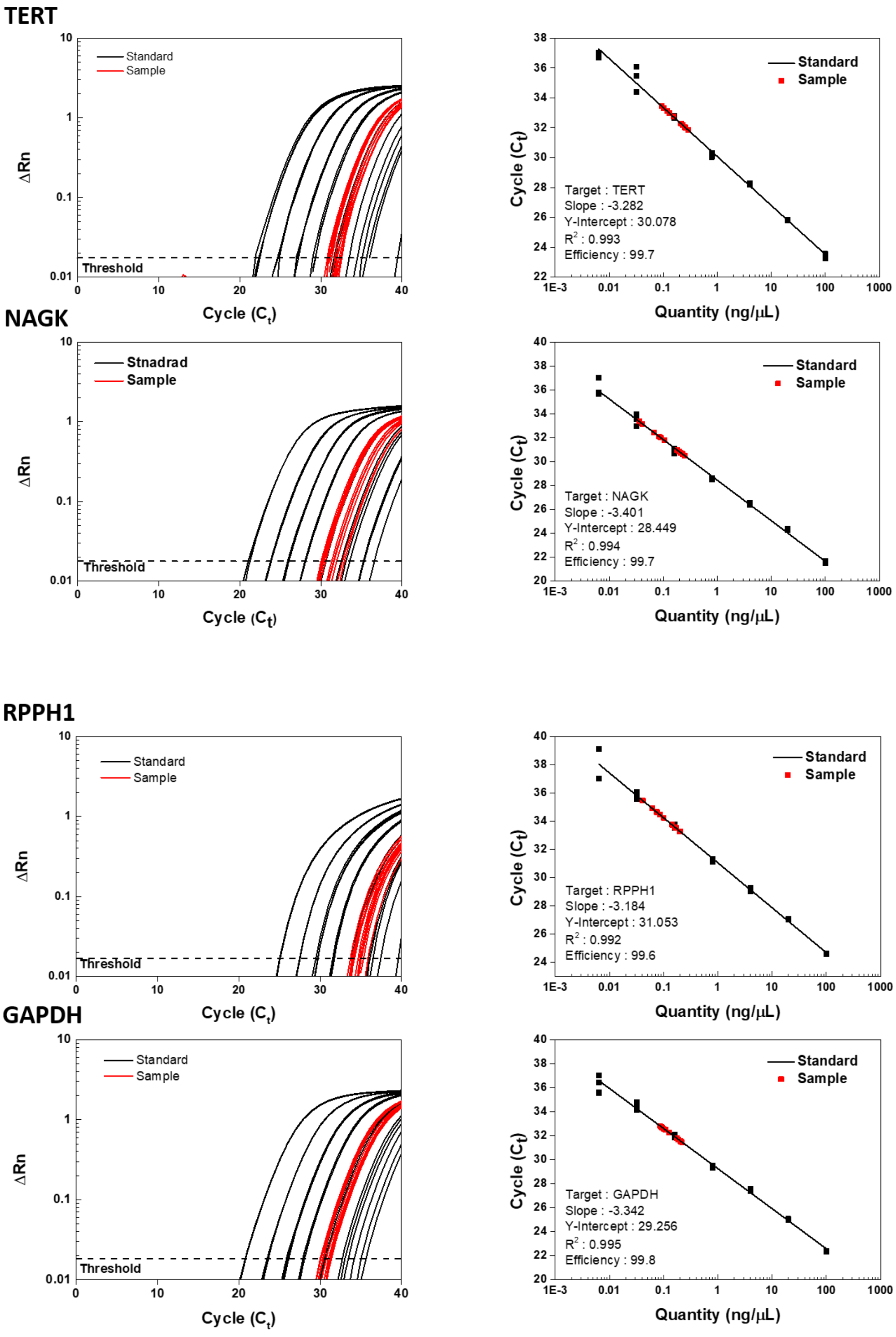

Fig. S2 Amplification plots and standard curves in the real-time PCR assay. Amplification plots (left) and standard curves (right) of *TERT*, *NAGK*, *RPPH1*, and *GAPDH*.

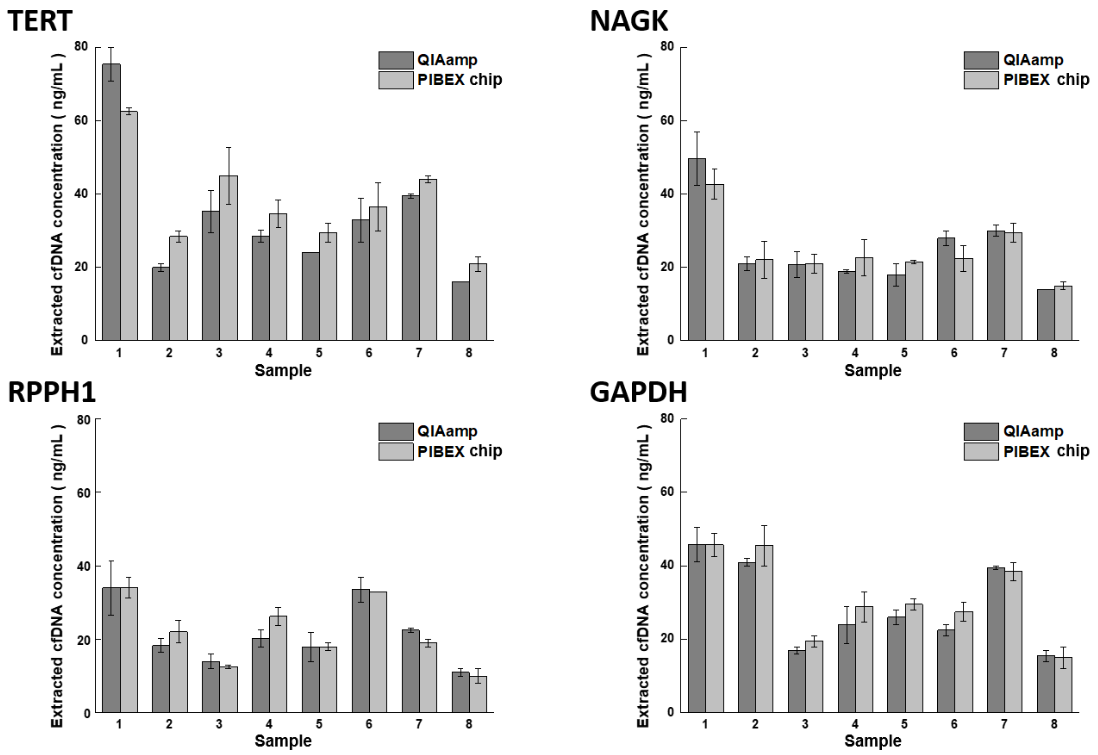

Fig. S3 Real-time PCR results with the four reference genes. Levels of *TERT*, *NAGK*, *RPPH1*, and *GAPDH* in cfDNA extracted by QIAamp or PIBEX chip.

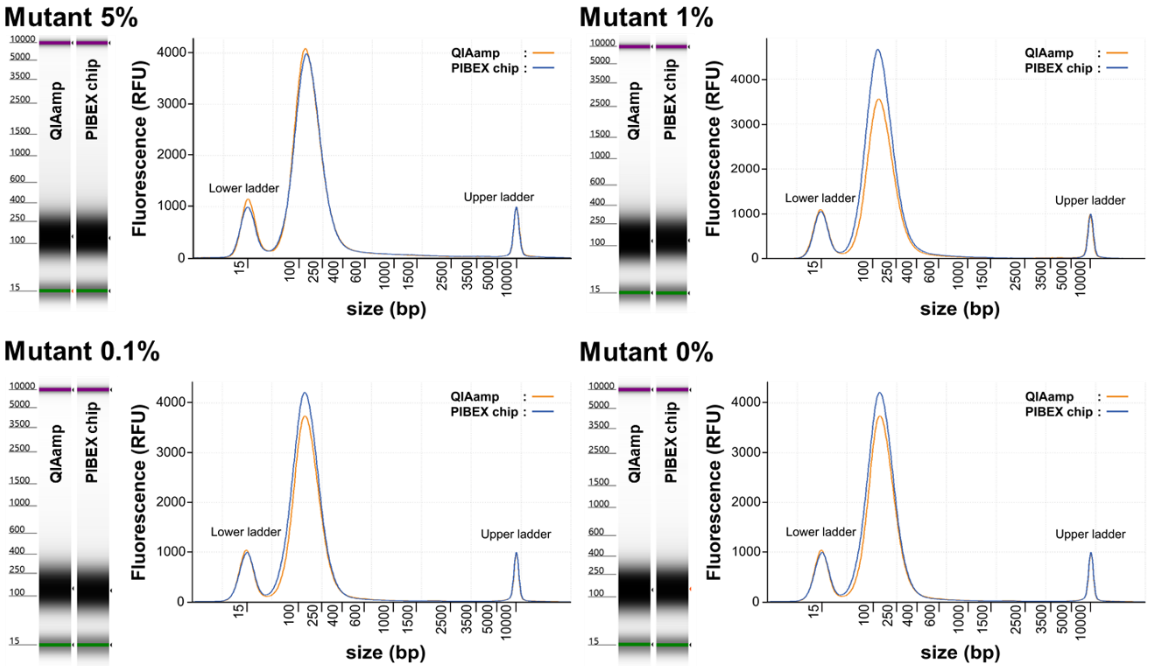

Fig. S4 Quality test of the cfDNA reference set. Quality test of the cfDNA reference set in synthetic plasma measured by microelectrophoresis.

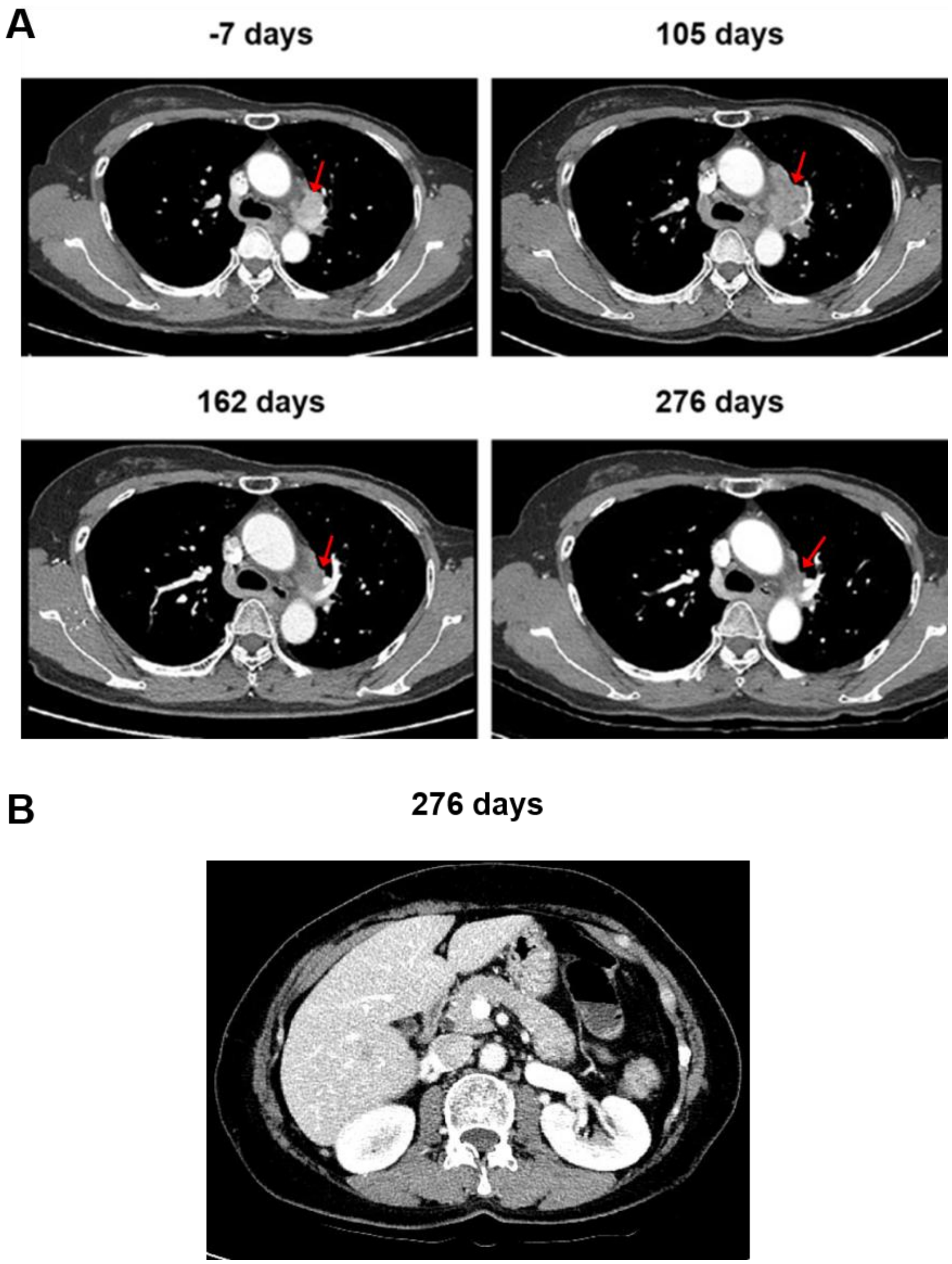

Fig. S5 CT images of *HER-2* type of breast cancer patient. A) Chest CT images from -7 days to 276 days. Red arrows indicate lymph nodes. B) Abdominal CT image at 276 days.

Table S1 Characteristics of study subjects.

Healthy controls for real-time PCR assay

**Characteristic**		**Value**
**Age, years**		
	Average	26.8
	Range	21–35
**Gender**		
	Male	1
	Female	7
**Blood type**		
	A+	1
	B+	1
	O+	6
	AB+	0

Clinical sample for ddPCR assay

**ID**	**Disease**	**Sex**	**Age**	**TKI type**	**TNM (initial)**
BP024	Breast cancer	Female	58	HER-2+	pT1cN1M0

Table S2 PCR assay information for the standard cfDNA sample.

**Gene Symbol / Assay name**	**Location**	**Primer/Probe sequence**	**Primer / probe (μM)**
180 bp Lambda DNA primer	3704 – 3884	F: CCTCACATAAATGCTACCAAAC	0.25
		R: TTCCAAGAAGGAGGCCATAGTC	0.25

Table S3 Real-time PCR assay information.

Gene Symbol / Assay name	Genome loci	Location	Primer/Probe sequence	Primer / probe (µM)	Probe fluorophore / quencher
TERT	5p15.33	NC_000005.9 (1253282.. 1295178, complement)	F: CCTCACATAAATGCTACCAAAC	0.9	FAM / BHQ-1
			R: TTCCAAGAAGGAGGCCATAGTC	0.9	
			P: AAGAAATGAACAGACCCATCCCCCAGG	0.25	
RPPH1	14q11.2	NC_000014.8 (20811230.. 20811570, complement)	F: GCGGAGGGAAGCTCATCAG	0.9	FAM / BHQ-1
			R: GGACATGGGAGTGGAGTGACA	0.9	
			P: CACGAGCTGAGTGCG	0.2	
GAPDH	12p13	NC_000012.11 (6643585.. 6647537)	F: AGGTTTACATGTTCCAATATGATTCCA	0.45	FAM / BHQ-1
			R: ATGGGATTTCCATTGATGACAAG	0.45	
			P: CCGTTCTCAGCCTTGACGGTGC	0.225	
NAGK	2p13.3	NC_000002.11 (71295408.. 71305998)	F : TGGGCAGACACATCGTAGCA	0.2	FAM / BHQ-1
			R : CACCTTCACTCCCACCTCAAC	0.2	
			P : TGTTGCCCGAGATTGACCCGGT	0.1	

Table S4 Allelic frequency verification data in the cfDNA reference standard set in synthetic plasma.

Gene	Variant	5%			1%			0.1%			0%		
		Expected AF (%)	WT copies	Mutant copies	Expected AF (%)	WT copies	Mutant copies	Expected AF (%)	WT copies	Mutant copies	Expected AF (%)	WT copies	Mutant copies
EGFR	L858R	5.0	93120	4768	1.0	103520	1104	0.10	105600	128	0	106720	8
EGFR	delE745-A750	5.0	54240	3328	1.0	66880	880	0.10	61440	72	0	62560	0
EGFR	T790M	5.0	47360	2568	1.0	56480	592	0.10	54400	88	0	55520	24
EGFR	V769-D770insASV	5.0	45552	2080	1.0	53920	528	0.10	53280	40	0	54560	0
KRAS	G12D	6.3	75520	4880	1.3	86720	1200	0.13	82400	136	0	82080	8
NRAS	Q61K	6.3	79200	5280	1.3	92160	1248	0.13	94400	128	0	87840	0
NRAS	A59T	6.3	90560	6192	1.3	110560	1600	0.13	105120	176	0	106080	16
PIK3CA	E545K	6.3	80160	5184	1.3	91520	1184	0.13	88320	120	0	88640	8

Table S5 Monitoring results of ctDNA in NGS analysis.

Sample	Gene	Nucleotide change (Amino acid change)	Allele frequency (Mutant/Total)	Exonic effect	Clinical significance
Baseline	*PIK3CA*	c.3140A>G (H1047R)	9.15% (14/153)	Missense variant	Pathogenic
	EGFR	c.2471G>A (G824D)	0.51% (10/1957)	Missense variant	Drug response
	NOTCH1	c.3249C>T	47.03% (111/236)	Synonymous variant	Benign
	APC	c.6363_6365delTGC (A2122del)	1.17% (3/256)	In-frame variant	Uncertain significance
420 days	*PIK3CA*	c.3140A>G (H1047R)	37.72% (195/517)	Missense variant	Pathogenic
	NOTCH1	c.3249C>T	59.71% (544/911)	Synonymous variant	Benign
	ATM	c.3077+4G>A	0.76% (6/785)	Intron variant	Conflicting interpretations of pathogenicity
	ALK	c.875G>A (R292H)	0.29% (9/3102)	Missense variant	Uncertain significance
570 days	*PIK3CA*	c.3140A>G (H1047R)	41.10% (179/436)	Missense variant	Pathogenic

Table S6 Genes analyzed by Axen Cancer Panel 1.

**SNV / InDel (88 genes)**	**Fusion (3 genes)**
ABL1, AKT1, AKT3, ALK, APC, AR, ATM, AXL, BRAF, BRCA1, BRCA2, CCND1, CDH1, CDK4, CDK6, CDKN2A, CEBPA, CSF1R, CTNNB1, DDR2, EGFR, ERBB2, ERBB3, ERBB4, ERG, ESR1, ETV1, ETV4, ETV5, EZH2, FANCA, FANCC, FANCF, FANCG, FBXW7, FGFR1, FGFR2, FGFR3, FGFR4, FLT3, FOXL2, GNA11, GNAQ, GNAS, HNF1A, HRAS, IDH1, IDH2, JAK1, JAK2, JAK3, KDR, KIT, KRAS, MAP2K1, MAP2K2, MAP2K4, MET, MLH1, MPL, MTOR, MYC, MYCN, NOTCH1, NPM1, NRAS, NTRK1, NTRK2, NTRK3, PDGFRA, PIK3CA, PIK3R1, PPARG, PTEN, PTPN11, RAF1, RB1, RET, ROS1, RUNX1, SMAD4, SMARCB1, SMO, SRC, STK11, TP53, VHL, WT1	*ALK, RET, ROS1*
